# Chronic Active Epstein–Barr Virus Disease

**DOI:** 10.3389/fimmu.2017.01867

**Published:** 2017-12-22

**Authors:** Hiroshi Kimura, Jeffrey I. Cohen

**Affiliations:** ^1^Department of Virology, Nagoya University Graduate School of Medicine, Nagoya, Japan; ^2^Medical Virology Section, Laboratory of Infectious Diseases, National Institute of Allergy and Infectious Diseases, National Institutes of Health, Bethesda, MD, United States

**Keywords:** chronic active Epstein–Barr virus, Epstein–Barr virus lymphoma, infectious mononucleosis, hemophagocytosis, DDX3X

## Abstract

Chronic active Epstein–Barr virus (CAEBV) disease is a rare disorder in which persons are unable to control infection with the virus. The disease is progressive with markedly elevated levels of EBV DNA in the blood and infiltration of organs by EBV-positive lymphocytes. Patients often present with fever, lymphadenopathy, splenomegaly, EBV hepatitis, or pancytopenia. Over time, these patients develop progressive immunodeficiency and if not treated, succumb to opportunistic infections, hemophagocytosis, multiorgan failure, or EBV-positive lymphomas. Patients with CAEBV in the United States most often present with disease involving B or T cells, while in Asia, the disease usually involves T or NK cells. The only proven effective treatment for the disease is hematopoietic stem cell transplantation. Current studies to find a cause of this disease focus on immune defects and genetic abnormalities associated with the disease.

## Introduction

Primary infection of adolescents and young adults often results in infectious mononucleosis with fever, lymphadenopathy, and sore throat (1). Additional signs and symptoms include splenomegaly, hepatomegaly, lymphocytosis, and liver dysfunction. Fever and lymphadenopathy usually resolve within 2 weeks after onset but can persist for a month, or in rare cases even longer. EBV is present in circulating B cells, and the level of EBV DNA is elevated in the blood for the first month of the illness. Both the innate immune response (especially NK cells) and the acquired immune response (virus-specific CD4 and CD8 cells) have a critical role in clearing the infection (2).

Initial control of EBV in healthy persons involves NK cells that can kill virus-infected cells (3, 4) and secrete IFN-γ, which inhibits B cell proliferation, and monocytes, which release chemokines in response to virus infection (5). A large clonal or oligoclonal expansion of CD8 cells is observed during infectious mononucleosis (6). Most CD8 cells are directed to lytic antigens initially, and these cells rapidly undergo apoptosis (7). These patients have modestly elevated antibodies to EBV lytic antigens as well as antibodies to the EBV nuclear antigens (EBNAs), including EBNA1.

Rare patients who become infected with EBV, or reactivate EBV, develop disease that does not resolve. Some of these patients develop fulminant infectious mononucleosis and die within days or weeks of primary infection. Others develop a more chronic course with persistent or intermittent infectious mononucleosis-like symptoms including fever, persistent lymphadenopathy, splenomegaly, and EBV hepatitis. These patients are unable to control EBV infection and have infiltration of tissues by EBV positive T, NK, or less often B cells. They have markedly elevated levels of EBV that persist in the blood. This entity is referred to as chronic active EBV (CAEBV) disease.

Some patients with CAEBV have been reported to have impaired NK cell (8) or T cell activity (9–13) against EBV-infected cells. In addition, reduced numbers of EBV-specific T cells have been described in patients with CAEBV disease (10). Unlike healthy persons with infectious mononucleosis, patients with CAEBV disease often have low numbers of EBV-specific CD8 cells (10). A recent study showed that patients with CAEBV or infectious mononucleosis have a decrease in the TCR-beta repertoire and expanded T cell clones in their peripheral blood compared with healthy carriers of EBV (14). Many have extremely high levels of antibodies to EBV lytic proteins and lack antibody to EBNA1 (13).

## CAEBV Definition and Features

Chronic active Epstein–Barr virus disease is usually defined as a chronic illness lasting at least 6 months, an increased EBV level in either the tissue or the blood, and lack of evidence of a known underlying immunodeficiency (15). Other authors, particularly when defining severe CAEBV disease, require both an elevated level of EBV in the blood as well as infiltration of tissues by EBV-positive lymphocytes (16). Recently, the duration of illness required for defining the disease has been shortened to 3 months (17). Former definitions required elevated levels of antibody to EBV viral capsid or early antigen in the blood (18); however, we have found that elevated levels of EBV DNA in the blood are more specific for CAEBV than elevated levels of EBV antibodies. Most laboratories now perform ELISA tests for EBV antibodies, and these are often less helpful than the previously used quantitative immunofluorescent assay using endpoint dilution of serum. It is important that DNA PCR is done using either whole blood or peripheral blood mononuclear cells, rather than plasma or serum which is much less sensitive for diagnosis of CAEBV disease.

**Figure 1:**
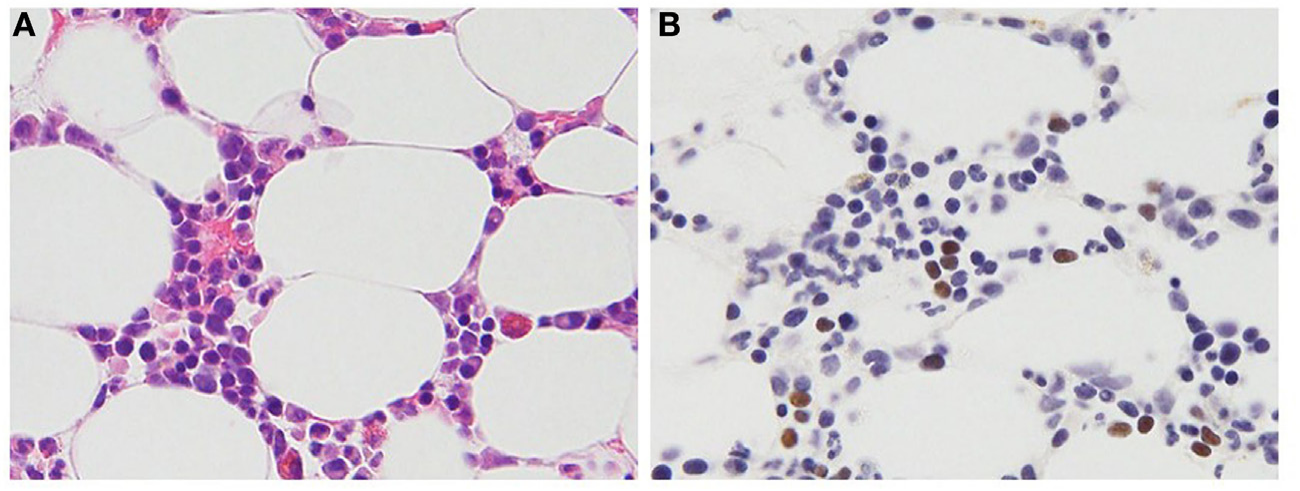
Histopathologic features of a 47-year-old female patient with T cell chronic active Epstein–Barr virus (EBV) disease. **(A)** Hematoxylin and eosin stain. Small- to medium-sized lymphocytes without significant atypia infiltrate the bone marrow clot. **(B)** EBV-encoded RNA *in situ* hybridization. The brown staining lymphocytes are positive for EBV RNA.

Chronic active Epstein–Barr virus disease was originally reported in children during primary infection, but in recent years, perhaps with increasing recognition of the disease, CAEBV disease has been reported in adults as well (19). CAEBV disease may be indolent with episodic fever, lymphadenopathy, and viral hepatitis followed by periods that are nearly asymptomatic; however, during these asymptomatic periods, the Epstein–Barr viral load remains very elevated. Alternatively, the disease can have a persistent or even fulminant presentation with death occurring in a few weeks. CAEBV disease is more frequent in Asians and in persons from South and Central America and Mexico. In these patients, EBV is predominantly present in T cells (Figure 1) or NK cells (20). In contrast, patients from the United States with CAEBV more often have EBV in B or T cells (16). In most healthy persons, EBV is latent in B cells; however, EBV can sometimes be detected in T and NK cells in the tonsils (21), and virus has been detected in T cells in persons with HIV (22) and other lymphoproliferative diseases (23, 24). At present, it is unclear how the virus enters T and NK cells; these cells do not express CD21, the EBV receptor.

Epstein–Barr virus gene expression in patients with CAEBV disease varies. There are four patterns of EBV gene expression, ranging from type 0 with no viral proteins expressed, although EBV EBV-encoded RNA and BART RNAs are expressed, to type 3 with all the latent viral proteins expressed including the EBV nuclear antigens (EBNAs) 1, 2, 3A–C, and LP, and latent membrane proteins (LMP) 1 and 2. Type 1 latency involves expression of EBNA1 and no other proteins; with type 2 latency, EBNA1, LMP1, and LMP2 are expressed. Patients with infectious mononucleosis have type 3 latency, whereas healthy EBV carriers have type 0 latency. Type 1 latency is seen in Burkitt lymphoma and type 2 in nasopharyngeal carcinoma, Hodgkin lymphoma, peripheral T cell lymphoma, angioimmunoblastic T cell lymphoma, and extranodal NK/T cell lymphoma (25). Most patients with CAEBV disease express a limited number of EBV latency genes. Although many patients have been reported with a type 2 latency pattern (26, 27), other patterns of EBV gene expression have also been reported, including type 3 (28). Thus, patients with T and NK cell CAEBV have a latency pattern that resembles that seen in EBV-positive T cell and NK cell lymphomas. These findings are consistent with a recent study showing that the cellular gene expression profile in patients with NK cell CAEBV is similar to that in NK cell lymphoma (29).

Epstein–Barr virus can be clonal, oligoclonal, or polyclonal in peripheral blood mononuclear cells of patients with CAEBV disease. Clonality for CAEBV has been based on PCR of the T cell receptor genes (for T cell CAEBV) or IgH genes (for EBV B cell disease) (16) or on the terminal repeat structure of the EBV genome (20). In one study of 17 patients, most patients had clonal EBV (27). Clonality does not necessarily indicate a worse prognosis (20).

Cells from patients with CAEBV can express both T-helper (TH1) (e.g., interferon-γ, IL-1β, IL-2) and TH2 (IL-4, IL-10, IL-13) cytokines (30). This failure to express a predominantly antiviral TH1 pattern has been referred to as an “unbalanced cytokine profile.” Patients with NK cell CAEBV disease were reported to have higher levels of IL-13 than those with T cell disease (27). Plasma levels of certain EBV microRNAs expressed from the BamH1 A fragment rightward transcript (BART) are higher in persons with CAEBV disease than in those with infectious mononucleosis or healthy controls (31). These findings suggest that these may be biomarkers useful for following these patients.

## Etiology

Initial reports suggested that CAEBV disease may be due to an unusual strain of EBV that results in lytic replication, but is impaired for transformation (32, 33), or a strain with a deletion in the viral genome (34). However, a subsequent study by one of these groups (35) showed that the unaffected father of the patient with CAEBV disease and some healthy controls had the same lytic strain of the virus as the patient with CAEBV, indicating that the unusual strain of EBV was not the cause of the disease.

Several features of CAEBV suggest that there is likely a genetic etiology. First, the impaired cytotoxic activity of T or NK cells (cited above) suggests that the disease could be due to an immunodeficiency. Second, the increased rate of the disease in Asians or natives of Central or South America suggests that the genetic background may play a role in the disease.

One study reported CAEBV in family members (36); however most recent cases do not describe multiple family members with the disease (16, 37). Studies have not found a consistent cause for CAEBV disease. Patients with meeting the definition of CAEBV B cell disease were subsequently found to have compound heterozygous mutations in perforin (38), compound heterozygous mutations in Munc13-4 (39), homozygous or compound heterozygous mutations in Munc 18-2 (39, 40), a heterozygous gain-of-function mutation in phosphoinositide 3-kinase p110δ (41), a mutation in MAGT1 (42), a mutation in GATA2 (43), and homozygous mutations in *CTPS1* (44). In each of the patients tested, EBV was predominantly in B cells. At present, no single genetic defect has been associated with a large proportion of patients with CAEBV disease.

Recent comprehensive genetic analysis by whole-exome sequencing showed that germline mutations are rare in CAEBV, but somatic driver mutations are frequently found in EBV-infected cells (45). Driver mutations including DDX3X and other genes associated with hematologic malignancies have been shown to accumulate in EBV-infected T/NK cells. In a case in which serial samples were obtained, clonal evolution of EBV-infected cells was confirmed with branching mutations in DDX3X. Mutations in DDX3X are frequently seen in Burkitt lymphoma and extranodal NK/T cell lymphoma (46, 47). These results indicate that serial acquisition of mutations in EBV-infected NK or T cells have the potential to result in transformation of the cells and may contribute to lymphomagenesis in this disease.

Although no single genetic defect has been identified in CAEBV disease, a positive association with human leukocyte antigen (HLA) A26 and a negative association with B52 were observed (48). Interestingly, both the A26 and B52 alleles are frequently seen in East Asia and Mexico, where the prevalence of the disease is high. Associations with HLA loci have been reported in other EBV-associated malignancies that show geographically distinct distributions (49, 50).

## CAEBV in the United States

In the largest series of CAEBV reported in the United States, EBV was often detected in B cells in tissues from patients, with cases of T and NK cell disease less common (16). The age of onset ranged from 4 to 51 years (mean 19 years). Patients with T cell disease were younger (mean age 7 years) than those with B cell disease (mean age 23 years). Lymphadenopathy and splenomegaly were the most frequent signs and symptoms, followed by fever, hepatitis, hypogammaglobulinemia, pancytopenia, hemophagocytosis, and hepatomegaly. Less common symptoms included pneumonitis, central nervous system disease, and periphery neuropathy. Some patients had B cell lymphopenia, others had reduced numbers of NK cells, and some had low numbers of both cells. Deaths were most often due to progressive EBV lymphoproliferative disease or opportunistic infections.

## CAEBV in Asia

T or NK cell CAEBV has a geographical predisposition, with most cases occurring in East Asians and some cases in Native American populations in the Western hemisphere (16). This distribution is analogous to that of extranodal NK/T cell lymphoma, also referred to as nasal NK/T-cell lymphoma. In Japan, nearly 60% of cases of CAEBV are T cell type, while 40% are NK cell type (37). EBV-infected T cells are variable: CD4^+^ T cells, CD8^+^ T cells, CD4^+^ and CD8^+^ T cells, CD4^−^ and CD8^−^ T cells, and γδ T cells have all been reported as the predominant cell type in individual patients with CAEBV. EBV-infected T or NK cells usually express cytotoxic molecules, such as perforin, granzyme, and T-cell intracytoplasmic antigen (TIA)-1 (51, 52), indicating that they have a cytotoxic cell phenotype.

The age at the onset of CAEBV in Asia ranged from 9 months to 53 years (mean, 11.3 years) (20). The signs and symptoms of CAEBV differ in frequency in the US and in Asia (Table 1). Typically in Asia, patients develop fever, hepatosplenomegaly, and lymphadenopathy; other common symptoms are thrombocytopenia, anemia, skin rash, diarrhea, and uveitis (20). The disease is sometimes complicated by hemophagocytic syndrome, coagulopathy, digestive tract ulcer/perforation, central nervous system involvement, myocarditis, interstitial pneumonia, multi-organ failure and sepsis (20). Interstitial pneumonia, calcifications in basal ganglia, and coronary aneurysms are occasionally seen without any symptoms. Some patients may have skin symptoms, such as hypersensitivity to mosquito bites and hydroa vacciniforme. Patients with severe mosquito bite allergy generally have EBV-infected NK cells, whereas those with hydroa vacciniforme often have EBV-infected γδ T cells (37). Patients with CAEBV sometimes develop T or NK cell neoplasms such as extranodal NK/T cell lymphoma, aggressive NK cell leukemia, and peripheral T cell lymphoma (37).

**Table 1:** Comparison of signs and symptoms of CAEBV disease in the US and Japan.

Sign or symptom	US (%)^a^	Japan (%)^b^
Lymphadenopathy	79	40
Splenomegaly	68	73
Fever	47	93
Hepatitis	47	67
Pancytopenia	42	NR
Hypogammaglobulinemia	42	NR
Hepatomegaly	32	79
Hemophagocytosis	32	24
Interstitial pneumonia	26	5
CNS disease	21	9
Neuropathy	21	NR
Rash	21	26
Hypersensitivity to mosquito bite	0	13
Hydroa vacciniforme	5	10

*NR, not reported*.

*^a^Ref. (16)*.

*^b^Ref. (20)*.

## Treatment and Prognosis

In the absence of treatment, patients with CAEBV develop progressive cellular and humoral immunodeficiencies and develop opportunistic infections, hemophagocytosis, multi-organ failure, or EBV-positive B, T, or NK cell lymphomas (53). CAEBV is refractory to antiviral therapy, interferon, intravenous immunoglobulin, and conventional chemotherapy and thus has a poor prognosis. Many other treatments have been tried including immunosuppressive agents such as cyclosporine or corticosteroids, autologous EBV-specific cytotoxic T cells, rituximab in the case of B cell CAEV, and the combination of bortezomib and ganciclovir. In some cases, these other treatments have resulted in transient reductions in systemic symptoms with improvement in laboratory abnormalities; however, the disease eventually returns and patients succumb to their disease if they do not undergo hematopoietic stem cell transplantation.

The survival of patients with T cell-type CAEBV is significantly lower, compared with that of patients with NK cell-type CAEBV (20). Hematopoietic stem cell transplantation alone is a curative treatment for the disease, although the incidence of transplantation-related complications is high (54, 55).

## Author Contributions

All authors listed have made a substantial, direct, and intellectual contribution to the work and approved it for publication.

## Conflict of Interest Statement

The authors declare that the research was conducted in the absence of any commercial or financial relationships that could be construed as a potential conflict of interest.
